# Rare Cutaneous Manifestations of Erdheim Chester Disease: A Case Report and Literature Review

**DOI:** 10.7759/cureus.40712

**Published:** 2023-06-20

**Authors:** Nikhil Vasandani, Jing Er Low, Yun Hui Liau, Alexander Ergun, Theogren Balakrishnan

**Affiliations:** 1 Department of Plastic and Reconstructive Surgery, University Hospital Galway, Galway, IRL; 2 Department of General Surgery, University Hospital Galway, Galway, IRL

**Keywords:** cd68+, braf mutation, diagnosis of rare cases, rare skin disease, skin nodule, pilomatrixoma, non-langerhans cell histiocytosis, erdheim chester disease

## Abstract

Erdheim Chester disease (ECD) is a rare and complex non-Langerhans histiocytic systemic disease that affects multiple organ systems, including the bones, heart, lungs, and central nervous system. Fewer than 1,000 cases have been reported in the medical literature and dermatological manifestations of the disease are rare but can provide valuable diagnostic clues for this challenging disease. The cutaneous manifestations of ECD can take many forms, including nodules, plaques, papules, and xanthomas. These lesions can occur on any part of the body and may be solitary or multiple. Cutaneous manifestations of ECD have been reported to occur in up to 20% of cases, but the true prevalence may be higher, as many cases may go undiagnosed.

We present the case of a 62-year-old gentleman with a history of ECD currently on vemurafenib who presented with multiple painless subcutaneous nodules on his back after an excision biopsy under local anesthetic revealed histological features of ECD. The objective of this case report is to raise awareness of ECD and its dermatological manifestations. Further research is warranted to better understand the pathogenesis and morphology of cutaneous involvement in ECD.

## Introduction

Erdheim Chester disease (ECD) is a rare non-Langerhans histiocytic systemic disorder of unknown etiology. It is characterized by the infiltration of different organs with CD-68 and CD-138 positive histiocytes surrounded by fibrosis [1]. It was originally described by Jakob Erdheim and William Chester back in 1930. Since then, fewer than 1,000 cases have been reported in the literature [1,2].

ECD typically affects adults between 40 and 60 years old with a male-to-female ratio of 3:1 [2]. It primarily affects the bones, lungs, heart, and central nervous system with clinical presentation ranging from constitutional symptoms such as weight loss and fatigue to organ-specific symptoms such as bone pain, lung disease, and seizures [1,3]. A recent literature review reported that cutaneous manifestations have been found in up to 20% of cases, with skin lesions such as xanthomas, nodules, and papules being reported in association with this condition [3].

The diagnosis of ECD involves identifying its characteristic histopathological features with correlation to patient clinical and radiological findings [3]. Cutaneous manifestations of ECD can pose a diagnostic challenge due to other various differential diagnoses but clinicians should consider a potential diagnosis of ECD when presented with a spontaneous case of unusual skin lesions in conjunction with unexplained systemic symptoms.

## Case presentation

We present a case of a 62-year-old gentleman with a history of ECD who was referred by dermatology to our plastic surgery service for excision of multiple painless, progressively enlarging subcutaneous nodules on the lower back which had been continuously persistent for the past seven years.

**Figure 1:**
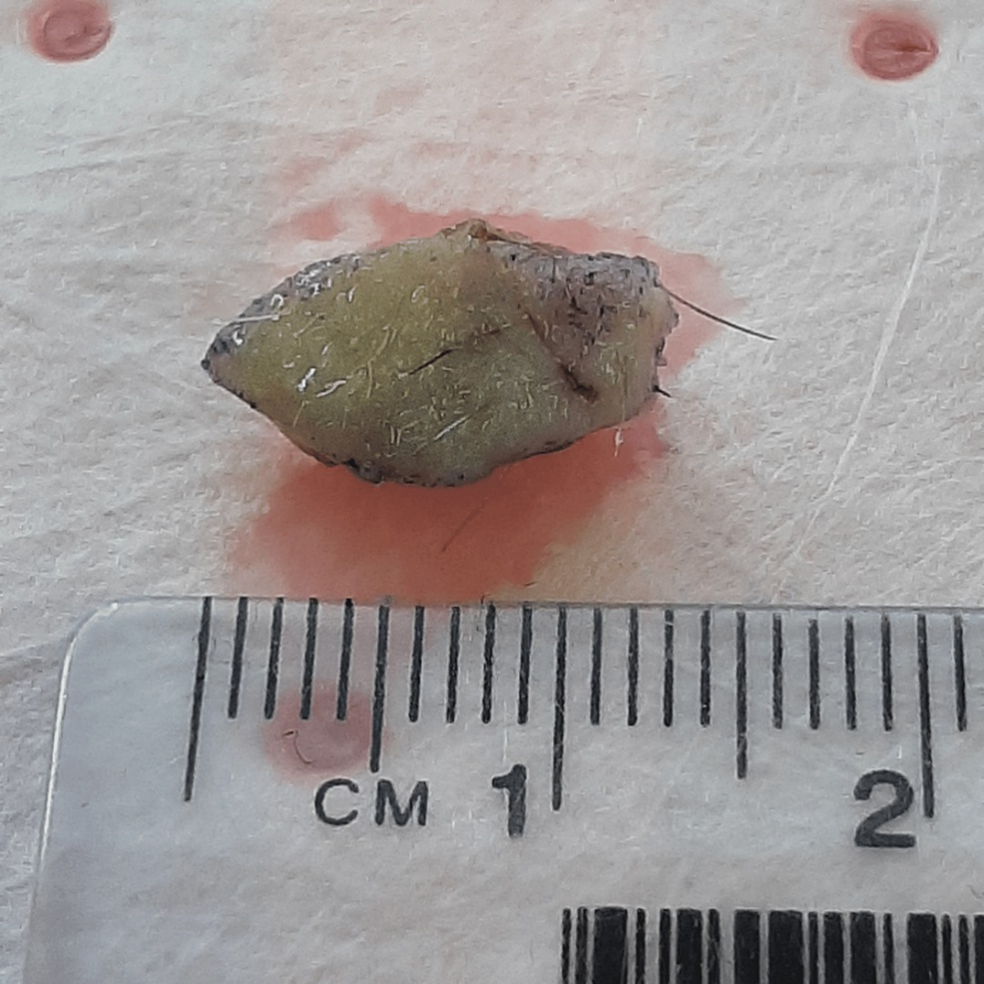
Lower back subcutaneous nodule gross specimen

The patient was diagnosed with ECD seven years prior to presentation and is currently being treated with BRAF inhibitor vemurafenib which has subsequently led to a significant improvement in his overall symptoms. On closer inspection, there were multiple, approximately 2 x 1 cm lumps on the lower back. The overlying skin was slightly erythematous but grossly normal. There were no acute signs of infection or inflammation. On palpation, masses were symmetrical, well-circumscribed, regular, firm, solitary, and subcutaneous in origin. The patient was asymptomatic and did not appreciate any tenderness upon palpation or manipulation. An excision biopsy was performed under 2% xylocaine with adrenaline local anesthetic without any complications and the sample was sent for histopathological analysis (Figure 1).

**Figure 2:**
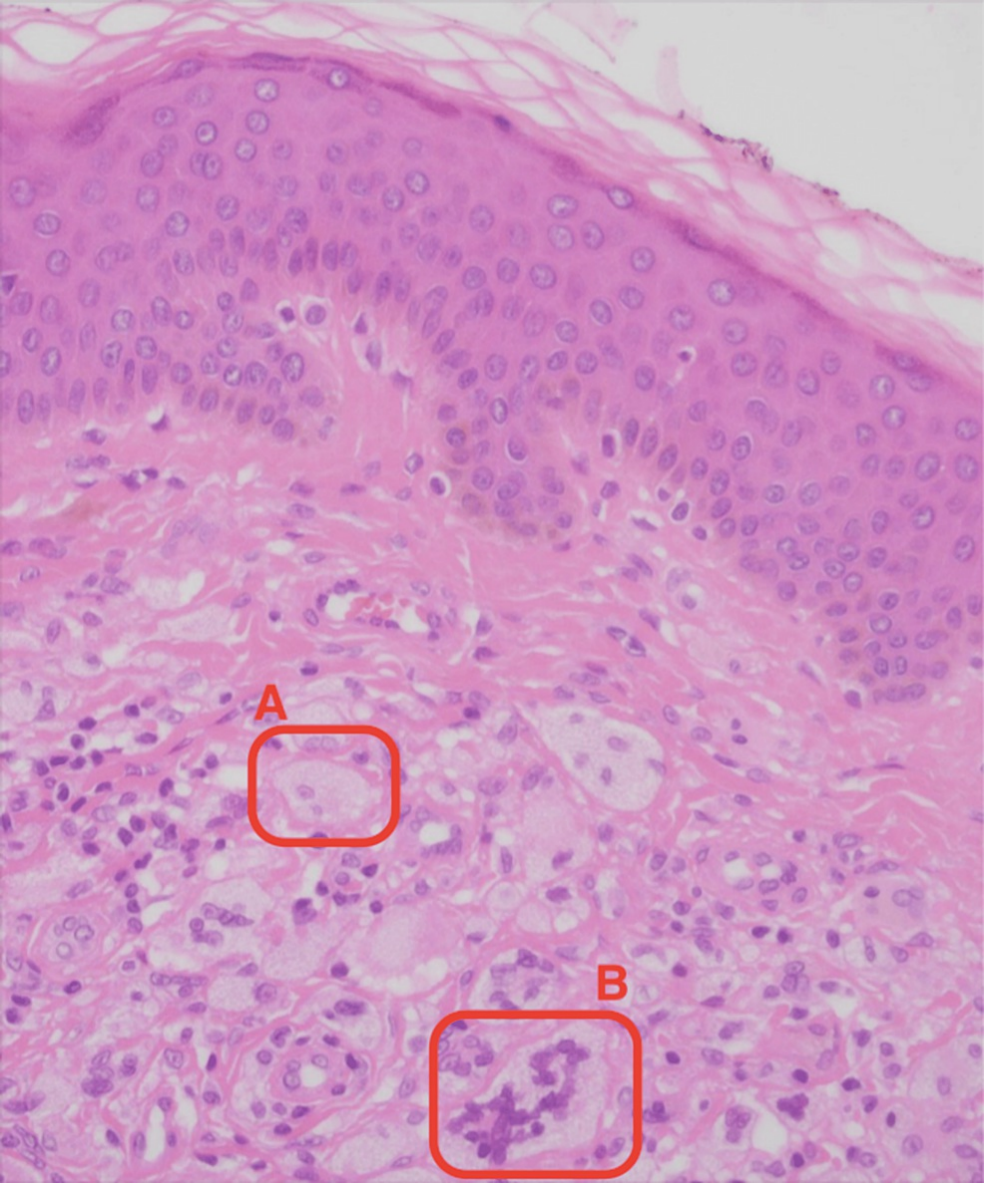
Foamy histiocytic cells (labelled A) and multi-nucleate giant cells (labelled B) seen on high power (40x) magnification

**Figure 3:**
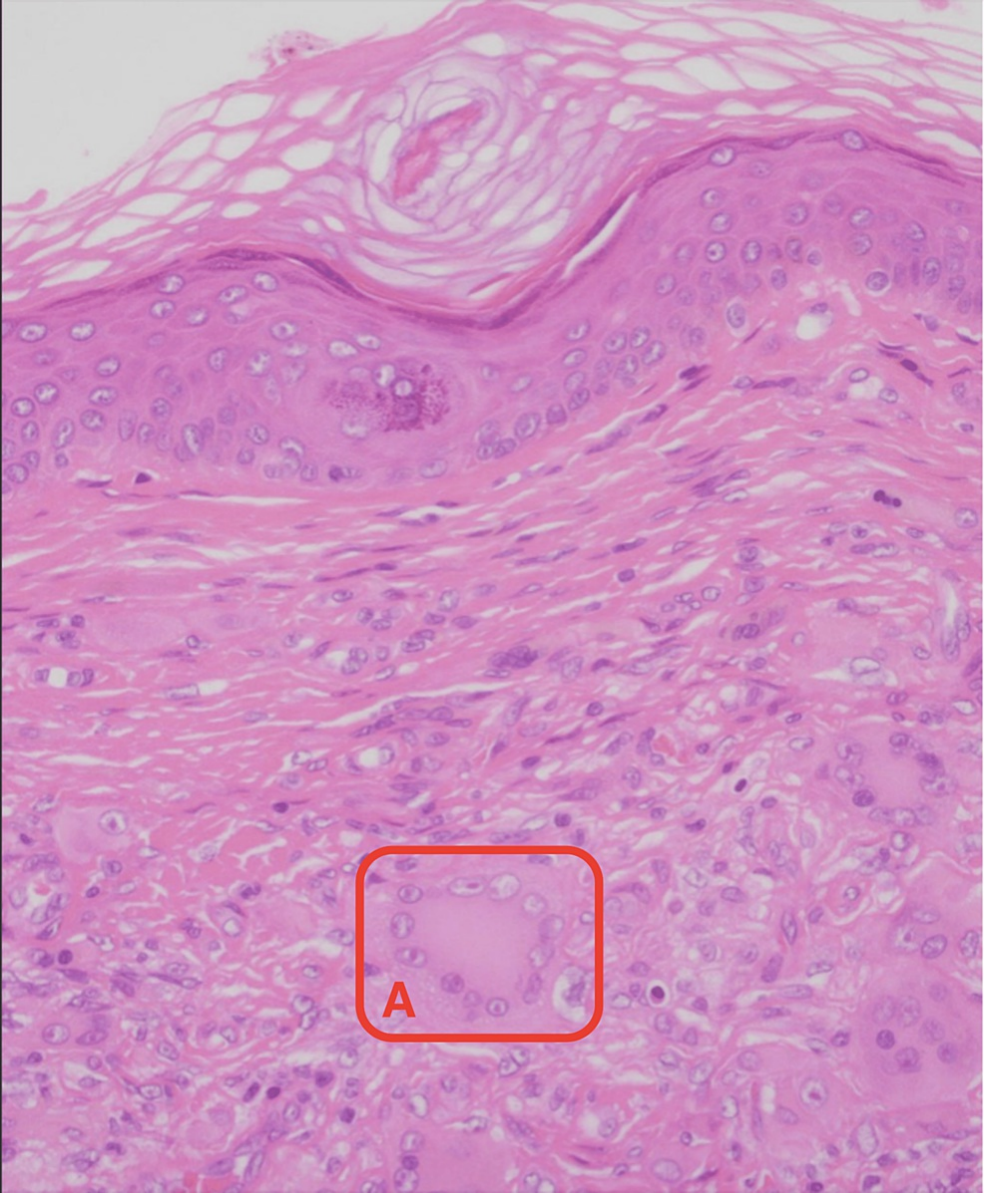
Touton giant cell (labelled A) seen on high power (40x) magnification

**Figure 4:**
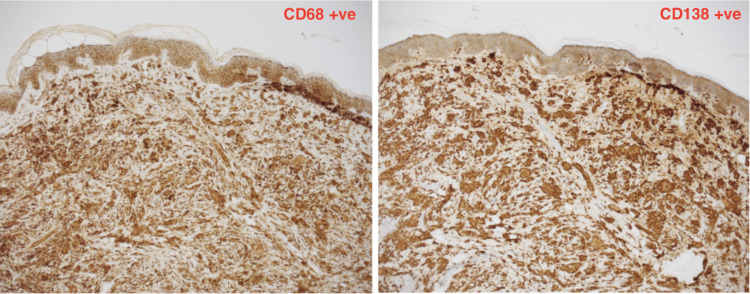
Immunochemistry analysis showing positivity for CD68 and CD138 confirming these are histiocytic cells

Gross examination of the lump revealed a 15 x 7 x 6 mm lesion. Histopathological examination revealed features consistent with ECD. Multiple foamy histiocytic cells, multi-nucleate giant cells (Figure 2) and touton giant cells (Figure 3) were appreciated. An immunochemistry analysis was performed which revealed CD68and CD138 positivity (Figure 4) confirming them to be histiocytic in origin. The lesion was excised completely with a 1 mm margin.

The patient had an uneventful postoperative course and was discharged home the same day. He continued on his treatment with vemurafenib and remained asymptomatic at follow-up visits.

## Discussion

**Figure 5:**
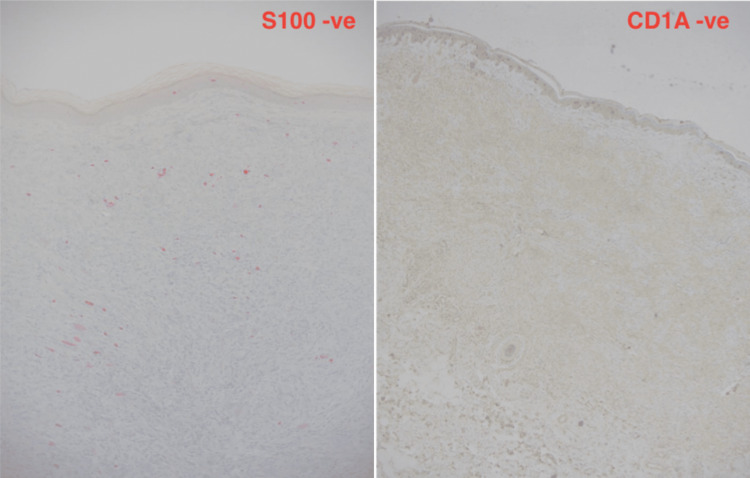
Immunohistochemistry analysis revealing S100 and CD1A negativity outruling RDD and LCH, respectively RDD: Rosai Dorfman disease, LCH: Langerhans cell histiocytosis

ECD commonly manifests as multifocal sclerotic lesions of the long bones. ECD is a histopathological diagnosis that is made by obtaining adequate tissue biopsies. Biopsies should demonstrate the characteristic histopathologic findings of lipid-laden xanthomatous histiocytes that typically have small nuclei and foamy cytoplasms and multi-nucleated giant histiocytes called Touton cells. In addition, there may be interspersed inflammatory cells and fibrosis [4]. For many patients, a biopsy of bone or another organ may be required to make a diagnosis. In addition, Langerhans cell histiocytosis (LCH) and Rosai Dorfman disease (RDD) have very similar histopathological findings to ECD. It is often a close differential and subsequently a common cause of ECD misdiagnosis. Immunohistochemical (IHC) tests analyzing S100 and CD1A markers are often performed to exclude RDD and LCH. In this case, CD1A and S100 were both negatively outruling LCH and RDD, respectively (Figure 5).

ECD is driven by somatic mutations of BRAF and/or other signaling molecules. No infectious or environmental cause of ECD has been identified in the literature, and there is no evidence to suggest that it is inherited [5]. BRAF V600E mutation has been detected in approximately half of ECD patients [6]. As a result, symptomatic patients presenting with evidence of organ dysfunction or CNS involvement are often treated with BRAF inhibitors like vemurafenib. Other therapies include MEK inhibitors, interferon alfa, glucocorticoids, or systemic chemotherapy. Asymptomatic patients who have no evidence of organ dysfunction or CNS involvement are usually observed relative to being treated immediately [7].

Clinical presentation is extremely varied given the systemic nature of the disease. Most patients have bony involvement at the time of diagnosis and the vast majority also have at least one extra-osseous site of involvement [8]. In our case, the patient was diagnosed with ECD seven years ago following a five-year stint of unexplained bone pain of the distal extremities and generalized constitutional symptoms of systemic disease including myalgia, unexplained fatigue, and weight loss. Subsequent x-rays revealed bilateral, symmetric osteosclerosis of the long bones (a radiological feature of ECD) and the patient ultimately underwent a bone biopsy, which revealed characteristic histopathological features of ECD [9]. Unfortunately, bone biopsies and x-rays of the aforementioned patient were performed in a different hospital and a different jurisdiction. Thereby, histopathologic and radiologic images could not be sourced.

In our case, seven years following diagnosis, the patient presented with multiple subcutaneous skin nodules in the lower back which after an excision biopsy and histopathological analysis revealed all the features of ECD classifying these lesions as dermatological manifestations. In addition, immunohistochemical analysis using CD68, CD138, S100, and CD1A stains was performed to exclude common differentials of ECD which include LCH and RDD. It was declared by the patient that these lesions were present seven years ago at the time of initial diagnosis but were not investigated.

A thorough literature review was performed on the cutaneous manifestations of ECD. Cutaneous manifestations of ECD are present in a quarter of patients and can take on various forms including xanthoma-like papules and periorbital xanthelasma [10,11].

A search on PubMed done in May 2023 using the terms, “Erdheim Chester disease OR Erdheim Chester syndrome” AND “skin OR cutaneous” yields over 140 articles. After filtering for English articles with abstracts and full text available, 98 studies were screened for relevance. One study was duplicated and 63 studies were deemed irrelevant in the abstract review. Thirty-four studies underwent full-text screening and 12 were excluded due to not having patients presenting with cutaneous manifestations of ECD. Data were then extracted from the remaining 22 studies. This included 15 case reports, five cohort studies, and two case series.

A single-center retrospective observational study performed in 2016 involving 40 patients with cutaneous manifestations of ECD revealed xanthelasma-like lesions (XLL) to be the most common cutaneous manifestation of ECD [10]. XLL can occur at various sites including the antecubital fossa, groin, breasts, axilla, eyelids, and neck [11]. Another observational cohort study published in 2017 involving 60 patients with ECD revealed periorbital xanthelasma as the most common cutaneous manifestation, being present in 33% of patients. Other skin lesions include juvenile xanthogranuloma, diffuse xanthoma, reticulohistiocytoma, and rashes resembling Langerhans cell histiocytosis. It was also noted that the upper trunk and extremities are the most involved region [12].

Chesset et al. showed that among 40 patients with cutaneous manifestations of ECD, skin lesions were the first reported symptom experienced in 30% of patients. The same study revealed 36% of those patients were diagnosed with ECD based on cutaneous biopsies [10]. Estrada-Veras et al. revealed that out of 60 patients with ECD, 10% of patients presented with cutaneous manifestations while 25% had cutaneous involvement [12].

ECD usually affects middle-aged patients. Estrada-Veras et al. showed that the mean age at diagnosis was 53 years old with the age range of 20-74 years old [12]. Chesset et al. showed the mean age of diagnosis to be 51, with patients ranging from 23 to 80 years old [10]. Thus, it is extremely rare for this to occur in children. Our literature review revealed only one case report involving a child [13]. This case reported a four-year-old boy with generalized maculopapular scaly lesions throughout the body. These were annular on the forehead and ulcerated on the upper eyelid. These subsequently developed central atrophy and sclerosis. Eventually, he was diagnosed via an excision skin biopsy [13].

There is a male predominance for both diagnosis of ECD and cutaneous manifestations of ECD. Chasset et al. showed that there is a male-to-female ratio of 2:1 [10], whereas Estrada-Veras showed a male-to-female ratio of 3:1 [12].

Prognosis

There is no known cure for ECD. Historically, the prognosis has been poor. However, the advent of targeted intervention and immunotherapy has drastically improved overall survival. In 1996, the five-year survival rate for ECD was 43%; however, according to a recent study, the survival rate has increased to 83%. It is hypothesized this is the outcome of recent therapeutic advancements.

As per the literature, the prognosis is dependent on the site of histiocytic infiltration and the general response to treatment [14]. One report suggested that older age, CNS involvement, digestive involvement, and high C-reactive protein (CRP) levels are associated with worse survival [14]. Another study reported that cardiovascular involvement was related to poor prognosis [15]. In 2011, Arnaud et al. reported that interferon-alpha (IFN-α) improved overall patient survival and increased five-year survival rates to 68% [16]. After BRAF-V600E mutations were observed in patients with ECD, it was reported that treatment with a BRAF inhibitor (vemurafenib) improved five-year survival rates to 79% with some patients achieving complete remission [9].

CNS involvement is a significantly poor prognostic factor. It is hypothesized that this is secondary to IFN-α resistance that develops in this patient cohort [16]. Moreover, a recent study revealed that CRP level at onset, as well as the drop in CRP levels after initial therapy, predict the patient outcome and survival [9]. Cardiovascular involvement of the disease confers a reduced response to treatment and an overall poor prognosis [15]. Approximately 75% of ECD patients suffer from cardiovascular manifestations and about 60% of them will perish due to cardiac complications with pericardial infiltration being the most frequent cardiac manifestation of ECD [15]. To improve the prognosis of patients with ECD, additional large-scale studies, more detailed analyses, and prospective studies of the pathophysiology of ECD are required.

ECD is a rare disease with an improving prognosis that harnesses a myriad of manifestations rendering it challenging to diagnose. As a result, delayed or erroneous diagnosis is very common. By understanding and being aware of the multiple other clinical manifestations of ECD, diagnosis can be made more accurately and differentials can be excluded more easily. Cutaneous features of ECD are common early manifestations that are usually underlooked and disregarded. We recommend that routine excision biopsies be performed on patients presenting with unexplained cutaneous lesions alongside features of systemic disease. This is a minimally invasive procedure that can yield adequate diagnostic material worthy of diagnosis of rare multi-system diseases such as this.

## Conclusions

In conclusion, we present an interesting case of a patient with a rare type of non-Langerhans histiocytic disease presenting multiple skin lesions on the lower back. This case report presents some of the cutaneous manifestations of ECD alongside its histopathological features, IHC findings, and a detailed literature review. This case teaches us the importance of multi-specialty involvement, the enigma surrounding some systemic disorders, and reminds us of the complexity of the human body. It highlights some of the atypical ways rare pathologies can present and how the skin can pose as a diagnostic clue and act as a presenting complaint for many of these patients. It reminds us of the vastness of the skin and how it can be the perfect playground for the presentation of many systemic disorders.
